# *COL4A1*-related autosomal recessive encephalopathy in 2 Turkish children

**DOI:** 10.1212/NXG.0000000000000392

**Published:** 2020-01-10

**Authors:** Ahmet Yaramis, Hanns Lochmüller, Ana Töpf, Ece Sonmezler, Elmasnur Yilmaz, Semra Hiz, Uluc Yis, Serdal Gungor, Ayse Ipek Polat, Pinar Edem, Sergi Beltran, Steven Laurie, *Aysenur Yaramis, Rita Horvath, Yavuz Oktay

**Affiliations:** From the Pediatric Neurology Clinic (A.Y.), Private Office, Diyarbakir, Turkey; Children's Hospital of Eastern Ontario Research Institute (H.L.), University of Ottawa, Canada; Division of Neurology (H.L.), Department of Medicine, The Ottawa Hospital, Canada; John Walton Muscular Dystrophy Research Centre (A.T.), Institute of Genetic Medicine, Newcastle University, UK; Dokuz Eylul University (S.H., E.S., E.Y., Y.O.), Izmir International Biomedicine and Genome Institute, Turkey; Faculty of Medicine (S.H., U.Y., A.I.P., P.E.), Department of Paediatric Neurology, Dokuz Eylul University, Izmir, Turkey; Faculty of Medicine (S.G.), Turgut Ozal Research Center, Department of Paediatric Neurology, Inonu University, Malatya, Turkey; CNAG-CRG (S.L., H.L., S.B.), Centre for Genomic Regulation, Barcelona Institute of Science and Technology, Spain; Universitat Pompeu Fabra (S.L.), Barcelona, Spain; Koc University (A*.Y.), School of Medicine, Medical Student, Istanbul, Turkey; Department of Clinical Neurosciences (R.H.), University of Cambridge School of Clinical Medicine, UK; Izmir Biomedicine and Genome Center (Y.O.), Dokuz Eylul University Health Campus, Turkey; and Faculty of Medicine (Y.O.), Department of Medical Biology, Dokuz Eylul University, Izmir, Turkey.

## Abstract

**Objective:**

This study presents the neurologic phenotypes of 2 brothers with a novel homozygous *COL4A1* mutation that was identified in a large Turkish consanguineous cohort of neurogenetic diseases.

**Methods:**

Whole-exome sequencing and bioinformatic analysis of consanguineous families with children affected by early-onset, neurogenetic disorders was performed using the RD-Connect Genome-Phenome Analysis Platform. We also performed clinical, EEG, and neuroimaging analyses in unaffected siblings and parents.

**Results:**

We have identified a homozygous missense mutation in *COL4A1* (p.Gly1278Ser, NM_001845.5:c.3832G>T) in 2 siblings affected by small vessel brain disease with periventricular leukoencephalopathy and ocular defects. Presenting symptoms included mild weakness, hemiparetic gait, pyramidal findings, and seizures, whereas their intellectual and behavioral functions were normal. Both parents and 5 of the siblings (3 boys and 2 girls) were heterozygous for the variant. They did not show any clinical or laboratory signs of small vessel disease.

**Conclusions:**

*COL4A1* has previously been associated with dominant small vessel disease of the brain and other organs, manifesting with high penetrance in heterozygous mutation carriers. Our findings provide evidence that *COL4A1*-related encephalopathy can be inherited in an autosomal recessive manner, which is important for counseling, prognosis, and treatment. Genotype-phenotype correlations remain to be established.

Type IV collagens are expressed in basement membranes only. Six different genes in humans encode 6 different alpha-chains (α1-α6), which form trimers with each other in 3 combinations: α1α1α2, α3α4α5, and α5α5α6. The classical type IV collagens, COL4A1 and COL4A2, are the major forms expressed during early development.^1^ The human *COL4A1* gene, located on chromosome 13 (13q34), is 158,199 bp long with 52 exons, and it encodes alpha-1 (α1) chain. COL4A1 protein contains a 27 amino acid signal peptide, a 7S domain at the N-terminal, a collagenous domain that forms a triple helix with other collagens, and a noncollagenous region (NC1) at the carboxy terminal.

*COL4A1*-related disorders are inherited in an autosomal dominant manner or occur de novo. The proportion of cases caused by a de novo pathogenic variant is estimated to be at least 27%–54%.^2,3^ Phenotypes include porencephaly type 1 (Online Mendelian Inheritance in Man [OMIM]# 175780), brain small vessel disease with or without ocular anomalies (OMIM# 607595), tortuosity of retinal arteries (OMIM# 180000), and hereditary angiopathy with nephropathy, aneurysms, and muscle cramps (HANAC) (OMIM# 611773).^4,5^ Intrafamilial and interfamilial variability are common findings in all *COL4A1*-related disorders.

Herein, we describe a homozygous missense *COL4A1* variant in 2 brothers, from a first-degree consanguineous Turkish family, both of whom have small vessel brain disease including small internal porencephaly, associated with eye defects such as retinal vessel tortuosity, Axenfeld-Rieger anomaly, cataract, and glaucoma. Similar to the other *COL4A1* cases in the literature, this particular *COL4A1* variant (NM_001845.5:c.3832G>T, NP_001836.3:p.Gly1278Ser) affects a glycine residue, interrupting the Gly-Xaa-Yaa amino acid repeat. However, in contrast to most of the previously reported glycine variants in *COL4A1*-related disorders, this variant causes defects that are inherited in an autosomal recessive manner.

## Methods

### Description of the study

Analysis of the family was performed as part of a large international genomic study on 200 consanguineous Turkish families that aims to determine the genetic burden of consanguineous marriages on neurogenetic diseases in Turkey by 3 centers in Turkey and 2 centers in the United Kingdom. Whole-exome sequencing (WES) was performed at the BROAD Institute of MIT-Harvard, and bioinformatic analysis was performed at the Centro Nacional de Análisis Genómico (CNAG)-CRG (Barcelona, Spain).

### Standard protocol approvals, registrations, and patient consents

The study was approved by the Dokuz Eylul University Clinical Research Ethics Committee (approval no. 302-SBKAEK 2016/03-01). Written consents of the patients and their parents were obtained. Written consent to publish photographs of the patients was also obtained.

### Clinical evaluation

Phenotypic studies included clinical evaluation, funduscopic examination, brain MRI, cerebral magnetic resonance angiography, abdominal ultrasonography, echocardiography, electrocardiography, urinalysis, and serum biochemical and hemogram levels of our affected patients and unaffected members of family. Written informed consent was obtained by one of the coauthors (A.Y.). Phenotypic data were collected with standard forms organized to include Human Phenotype Ontology terms, and data were entered into the PhenoTips database.

### WES and validation

WES was performed on DNA obtained from probands 2 and 3, and both parents, at the Broad Institute of MIT/Harvard. Libraries from DNA samples (>250 ng of DNA, at >2 ng/μL) were created with an Illumina exome capture kit (38 Mb target) and sequenced (150 bp paired-end reads) to cover >90% of targets at 20× and a mean target coverage of >80×. Sample identity quality assurance checks were performed on each sample. The exome sequencing data was demultiplexed, and each sample's sequence data were aggregated into a single Picard BAM file.

### Detection of *COL4A1* variant and segregation analysis

Sequencing data were processed at the CNAG, Barcelona, and data analysis was performed on the RD-Connect Genome-Phenome Analysis Platform^6^ using standard filtering criteria for rare diseases, including minor allele frequency <0.01, variant effect predictor = mod/high, and Combined Annotation-Dependent Depletion (CADD) >20. The *COL4A1* variant (NM_001845.5:c.3832G>A, NP_001836.3:p.Gly1278Ser) was tested by Sanger sequencing in both affected siblings, both parents and 6 unaffected siblings.

### Data availability

WES data were analyzed on the RD-Connect Genome-Phenome Analysis Platform, and anonymized data will be shared with any qualified investigator on approval of official request.

## Results

### Phenotypic evaluation

#### Case 1

**Figure 1:**
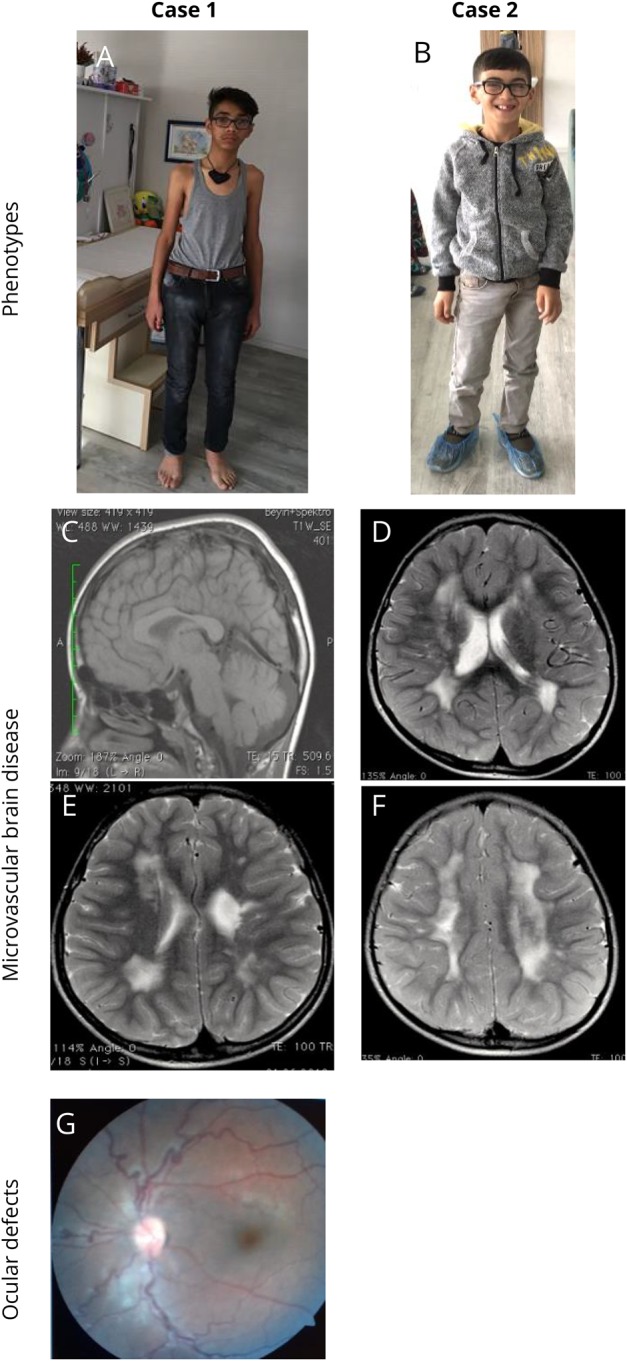
Selection of phenotypes, brain magnetic resonance images, and ocular defects of 2 cases (A) Case 1 (homozygous missense [p.Gly1278Ser] in *COL4A1*), aged 16 years at the last follow-up, had good academic skills with a marfanoid appearance, mild hemiparesis, partial seizures, and ocular defects. EEG was abnormal. Brain MRI findings showed that diffuse PVL with left ventricular enlargement with small porencephaly and thinning of the body part of the corpus callosum were stable even after 6 years. (B) Case 2 (homozygous missense [p.Gly1278Ser] in *COL4A1*), aged 10 years at the last follow-up, had good academic skills with mild hemiparesis and ocular defects. EEG was normal. Brain MRI findings showed that PVL with right mild ventricular enlargement was stable even 6 years later. (C) T1-weighted sagittal image of case 1, showing thinning of the body part of the corpus callosum. (D) T2-weighted axial image of case 2, showing diffuse PVL with right mild ventricular enlargement. (E) T2-weighted axial image of cases 1, showing diffuse PVL with left ventricular enlargement and small porencephalic cyst. (F) T2-weighted axial image of cases 2, showing diffuse bilateral deep white matter leukoencephalopathy. (G) Ophthalmoscopic examination of case 1 showing hypoplastic optic discs and tortuosity of retinal veins. PVL = periventricular leukoencephalopathy.

A 16-year-old boy was admitted at age 8 years. He had mild hemiparesis on the right side that started at age 8 years (figure 1A). His parents noticed this complaint when he was walking independently at age 18 months. He had a history of partial epilepsy with reduced consciousness that started at age 1 year and incompletely controlled by oral Na^+^ valproate treatment.

He had seizures usually twice a month, typically complex partial seizures or complex partial seizures evolving to generalized seizures localized to the right side. The patient was previously followed up with a diagnosis of perinatal hypoxic-ischemic sequelae at another center. His seizures were usually prolonged (>5 minutes) and sometimes lasted for about half an hour, which was compatible with status epilepticus.

He was born at home by spontaneous vaginal delivery with the help of a midwife without complication. Birth weight, head circumference, and height were unknown; therefore, the Apgar score could not be calculated. The mother had neither related significant medical history nor regular antenatal medical follow-up. Fetal movements during the antenatal period were normal.

His early motor development was normal, except having mild hemiparesis that was noticed at age 18 months. The patient's hand preference during the first 2 years is not known. The patient started walking without support at age 18 months. His social and language milestones were normal. At age 6 months, bilateral cataract was noticed. He was operated for cataract and glaucoma, and prophylactic glaucoma treatment was started.

His initial physical examination revealed a marfanoid appearance with disproportionately long arms and legs compared with the trunk. However, he had no signs of wrist (Walker) or thumb (Steinberg), or joint hypermobility or arachnodactyly, that are typical signs of Marfan syndrome. Cranial nerve examinations and eye movements in all directions were normal. He was using hypermetropic glasses. Ophthalmologic examination revealed bilateral megalocornea, trabeculodysgenesis, nebular corneal opacity, hypoplastic optic discs, and tortuosity of retinal venous vessels, findings that were suggestive of a congenital cataract and glaucoma (figure 1G). Neurologic examination revealed mild unilateral weakness with hemiparetic gait, increased reflexes, and Babinski sign on the right. He was making good academic progress at school and communication with the social environment.

His whole blood and routine biochemistry analyses with serum creatine kinase levels (80 units/L) were normal. EEG examination revealed frequent and irregular pathologic high-voltage theta- and alpha-wave groups with multifocal sharp and spike discharges in the left hemisphere. Brain background activity was normal.

Brain MRI of patient 1 showed diffuse periventricular leukoencephalopathy (PVL) with left ventricular enlargement, small porencephalic cysts, and thinning of the body part of the corpus callosum (figure 1, C and E). Brain MRI analysis was performed twice, at ages 3 and 9 years, and no differences were seen between the 2 MRI analyses. Brain MRI angiography analysis was normal at age 9 years.

His epileptic seizures were consistent with complex partial seizures evolving to generalized seizures according to the International League Against Epilepsy 1989 classification. Appropriate oral oxcarbazepine was introduced instead of sodium valproate. After the treatment, no seizures were observed during the 6-year follow-up of the patient.

In the following years, further diagnostic tests (serum levels of homocysteine, B12 vitamin, folic acid, lactate, pyruvate, biotinidase, and analyses of serum quantitative amino acids, acylcarnitines by tandem mass spectrometry, urine organic acid, serum arylsulfatase enzyme, and mitochondrial DNA sequence) were normal. Cardiac examination, echocardiogram, ECG, urine microscopic examination, and renal ultrasound were normal. His blood pressure and respiratory function were normal. The patient had a stable clinical course, and there were no complaints such as stroke, muscle cramps, and migraine-type headache.

#### Case 2

A 10-year-old brother was admitted with left-sided hemiparesis at age 2 years. This complaint was first noticed at age 16 months, when he began walking without assistance. He had no history of epileptic seizures. This case has also been followed up with a diagnosis of cerebral palsy due to perinatal hypoxic-ischemic encephalopathy in another center. The patient was born at term, with a natural delivery with the help of a midwife at home without complication. His Apgar score was not known. Birth weight, head circumference, and height were unknown.

Cranial nerve examinations and eye movements in all directions were normal. There was no ptosis, and he used glasses for hypermetropia. The patient's eye examination indicated mild depth in the anterior camera, as well as mild fibrils and spots in front of the lens of both eyes, which was compatible with the preliminary finding of glaucoma. The patient's first neurologic and physical examination revealed mild weakness with hemiparetic gait that was more prominent in the leg, increased reflexes, Babinski sign on the left.

Brain MRI showed diffuse PVL with right mild ventricular enlargement (figure 1, D and F). Brain MRI analysis was performed when the patient was aged 3 and 8 years. There were no differences in MRI findings that were performed with 5-year interval. Brain MRI angiography analysis was normal at age 8 years. He was making good academic progress at school and communication with the social environment, like his affected brother.

Parents are first cousins; they have 8 children in total; 3 daughters (13 years old, 20 years old, and 23 years old) and 3 sons (18 years old, 19 years old, and 22 years old) are unaffected. They did not have any complaints such as cerebral stroke, migraine headache, muscle cramps, heart, kidney, or any eye disease. There were no similar complaints in the first-degree relatives of parents either.

Cardiac examination, echocardiography, ECG, and renal ultrasound evaluations were normal. Funduscopic, cardiac, renal, and cerebral MRI evaluations and serum creatine kinase levels were normal in all unaffected family members including their parents. Of note, none of the siblings or the parents were clinically affected or showed any sign of glaucoma.

### Genetic analyses

**Figure 2:**
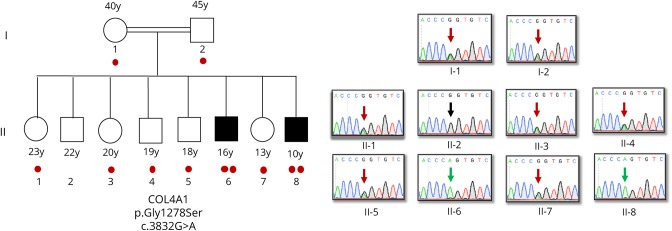
COL4A1 NM_001845.5:c.3832G>A p.Gly1278Ser variant segregates with disease Both parents and 5 of the 6 unaffected siblings are heterozygous for the variant, while both affected cases are homozygous. One unaffected sibling does not carry this variant. Number of “red dots” indicates the number of *COL4A1* c.3832G>A alleles for each family member.

**Figure 3:**
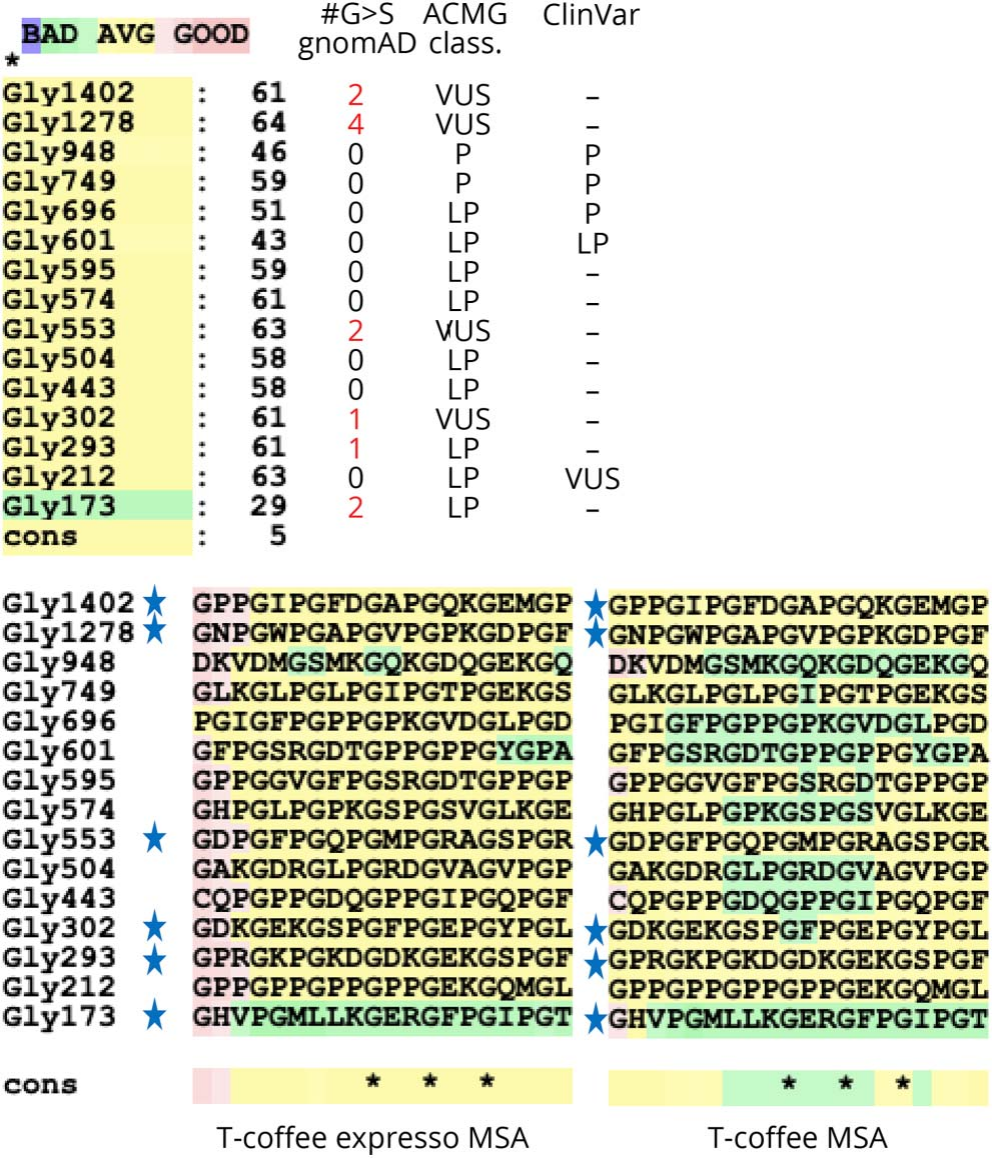
Multiple sequence alignment of sequences flanking glycines in the triple-helix region (THR) of COL4A1 that are known to be affected by Gly-to-Ser substitutions T-Coffee Expresso and T-Coffee alignments show higher similarity between those that flank a glycine with at least 1 nonpathogenic Gly-to-Ser substitution according to gnomAD (indicated by “star” sign). Number of heterozygotes in gnomAD, ACMG classification, and ClinVar status of variants are indicated next to T-Coffee Expresso similarity scores. ACMG = American College of Medical Genetics; gnomAD = Genome Aggregation Database; MSA = Multiples Sequence Alignment.

WES of the 2 affected brothers and their parents identified a novel homozygous missense variant (NM_001845.5:c.3832G>A; NP_001836.3:p.Gly1278Ser) in exon 43 of *COL4A1* (chr13:110169673, GRCh38.p12). Both affected siblings were homozygous, while both parents were heterozygous for the variant (figure 2). The variant is predicted to be pathogenic by MutationTaster, PolyPhen2, and Sorting Intolerant From Tolerant and has a CADD score of 28.2. Although the variant is defined in the Single Nucleotide Polymorphism database with identifier rs757453900, it is missing from the ClinVar database. The allele frequency is 0.000001592 according to the Genome Aggregation Database (gnomAD), with no homozygotes, but 4 heterozygotes out of 251,314 allele counts of healthy controls.^7^ The variant was missing from a cohort of 1,182 ethnically matched Turkish control individuals (TUBITAK MAM-GMBE data set).^8^ Gly1278 is a highly conserved residue (GERP = 5.47) and Gly1278Ser mutation interrupts the Gly-Xaa-Yaa amino acid repeats, similar to the previously reported pathogenic *COL4A1* variants that affect glycine residues in Gly-Xaa-Yaa repeats.^9^ Multiple sequence alignment of the protein sequence flanking Gly1278 to those regions flanking the other reported Gly-to-Ser variants in the COL4A1 triple-helix region (THR) does not point to any obvious differences that might explain the recessive inheritance of the associated pathologies (figure 3). However, it is intriguing that sequences flanking glycines with Gly-to-Ser variants in gnomAD show higher sequence similarity to each other compared with the others.

## Discussion

The spectrum of *COL4A1*-related disorders includes small vessel brain disease of varying severity including porencephaly, variably associated with eye defects (retinal arterial tortuosity, Axenfeld-Rieger anomaly, and cataract) and systemic findings (kidney involvement, muscle cramps, cerebral aneurysms, Raynaud phenomenon, cardiac arrhythmia, and hemolytic anemia).^5^

The inheritance of *COL4A1*- and *COL4A2*-related disorders is dominant: In a recent study, the authors reported 44 new patients, 24 due to de novo mutations; they also calculated the number of previously reported *COL4A1* and *COL4A2* mutations to be 63 and 6, respectively.^3^ Eight of 44 *COL4A1/COL4A2* mutations associated with epilepsy were de novo. However, 24 of them were of unknown inheritance. Therefore, the proportion of cases caused by a de novo pathogenic variant can be estimated to be at least 18%–54%, whereas another study suggested it to be at least 27%.^2^ Of interest, incomplete penetrance of *COL4A1* mutations was reported,^3^ and the authors suggested that it may contribute to increased disease severity over generations. Similarly, to explain the phenomenon of the same *COL4A1* variant causing disease in offspring but not in the parents, it was postulated that *COL4A1* mutations should be regarded as risk factors that interact with modifiers to cause disease. However, the family reported here with both affected individuals being homozygous and 5/6 unaffected siblings being heterozygous cannot simply be explained by incomplete penetrance or generational gradient in phenotype.

In this study, we have identified a missense variant in *COL4A1* (p.Gly1278Ser) that segregated in a clearly recessive trait with perinatal cerebral hemorrhage and diffuse PVL with ocular defects among 2 brothers, 6 unaffected siblings, and their parents.

Initially, cranial MRIs of the cases were reported to be consistent with leukodystrophy or perinatal hypoxic-ischemic encephalopathy by 3 different neuroradiologists at the same time. However, the clinical and radiologic findings of the patients were similar. Moreover, their parents were first-degree relatives, and there was no history of perinatal complications in 2 cases. Therefore, possibility of neurogenetic disease was considered.

The most abundant type IV collagens COL4A1 and COL4A2 both have collagenous triple-helical domains characterized by repetition of Gly-X-Y motifs, where X and Y are variable amino acids. Starting at the noncollagenous (NC1) domains, 2 molecules of COL4A1 and 1 molecule of COL4A2 assemble together to form a trimer, namely α1α1α2 (IV) collagen.

Stability of this trimer is dependent on intermolecular interactions at the THR and glycine residues are critical to preserving the trimer structure. By impairing heterotrimer biosynthesis, substitutions of the glycine residues are the most frequent pathogenic mutations in *COL4A1*- and *COL4A2*-related disorders, and all of the missense mutations in the THR of both proteins affect glycine residues.^10,11^ Moreover, 66% (39/59) of the reported glycine mutations in THR are conversion of glycine to charged amino acids (glutamic acid, arginine, and aspartic acid), and only 16% (8/59) of pathogenic missense mutations are Gly-to-Ser substitutions.^3^ Importantly, glycine mutations on 1 allele are usually expressed and make 50% of the monomers. Through the assembly process, those mutated monomers disturb around 90% of the heterotrimers, explaining their dominant negative effect. In addition, among the “benign” and “likely benign” variants of *COL4A1* reported in *ClinVar*, there is only 1 missense mutation (rs150182714, p.Gly332Arg) that affects glycines in the THR, underscoring the mutational intolerance of glycine residues in this critical region^12^ and making Gly1278Ser substitution an extremely rare example. Of note, well-known examples of recessively inherited collagen mutations are observed in *COL6A2* that cause Bethlem myopathy, Ullrich congenital muscular dystrophy, and congenital myosclerosis; however, none of the recessive mutations in *COL6A2* are glycine substitutions.^13^ It is possible that the sequence flanking Gly1278 has a structural or functional property that causes tolerance for glycine missense mutations. Such context-specific effects were reported for *COL6A3*.^13^ However, comparison of the sequence context of other pathogenic Gly-to-Ser mutations in *COL4A1* to that of Gly1278 does not provide an obvious explanation to the unique recessive inheritance pattern (figure 3). Yet, higher sequence similarity of “tolerant” Gly-to-Ser substitutions to each other compared with other Gly-to-Ser substitutions implies a structural and/or functional basis that remains to be explored. It is possible that Gly1278Ser variant interferes with the posttranslational processing of the monomer and reduces its interaction with wild-type monomers, thereby allowing formation of wild-type trimers in the heterozygous state. Further molecular studies are required to test this hypothesis.

Others did not observe a correlation between the severity of the disease and the location of the mutations.^3^ On the other hand, phenotypic heterogeneity for *COL4A1* mutations was reported even on a pure mouse genetic background, likely due to variation in the severity of stress factors.^14^ Considering the consistency in our pedigree, it is possible that the recessive inheritance pattern could be due to a favorable combination of protective modifiers that alleviates or reduces the effects of stress factors on basement membranes. This hypothesis is in line with the previously proposed “risk factor model.”^2^

Before 2005, congenital porencephaly on CT or MRI had usually been considered as the result of an external insult such as traumatic or hypoxic-ischemic birth injury. With the generation of mouse models of *COL4A1*-related disorder,^14,15^ *COL4A1* mutations were reported as the primary cause of hereditary porencephaly with an autosomal dominant inheritance.^2^

Over the years, variants in *COL4A1* have been identified in a wide range of diseases, including porencephaly,^16^ familial and sporadic small vessel disease and hemorrhagic stroke,^15,17,18^ leukoencephalopathy,^19^ HANAC syndrome,^10^ Walker-Warburg syndrome,^21^ and isolated ophthalmologic anomalies.^19–21^ *COL4A1*-related disorders represent a single disease with great variability in expressivity and age at onset. The complaints of our 2 brother cases were noticed by the parents in the their first 18 months of life when they started to walk independently. Of note, affected brothers are 2 of the 3 youngest of the 8 siblings. Although we cannot rule out the possibility of parents and/or unaffected siblings being presymptomatic, absence of similar complaints within the grandparents or cousins argues against this possibility.

In the literature, previously reported patients demonstrate severe clinical manifestations including porencephalic cysts on brain MRI as the most frequently reported finding, seizures, developmental delay, intellectual disability and behavioral abnormalities, microcephaly, and motor abnormalities on neurologic examination, with involvement of both abnormal pyramidal and extrapyramidal signs.^3^ In this study, cases 1 and 2 showed diffuse PVL with left ventricular enlargement with small porencephalic cysts, thinning of the body part of the corpus callosum, and diffuse PVL with right mild ventricular enlargement in MRI analyses, respectively. Although our cases had the pyramidal findings, their intellectual and behavioral functions were quite good. Their neuromotor developmental skills were normal, and neither one had microcephaly. Case 1 had epilepsy since age 1 year.

In this study, we describe a rare case of autosomal recessively inherited homozygous missense *COL4A1* variants in 2 brothers, parents of whom are first-degree cousins, with small vessel brain disease including small internal porencephaly, associated with eye defects such as retinal vessels tortuosity, Axenfeld-Rieger anomaly, cataract, and glaucoma. Enlarged clinical spectrum and an unusual inheritance pattern for *COL4A1* variants will improve the effectiveness of targeted screening of the *COL4A1* gene and appropriate clinical management of the patients.

## Glossary

CADD: Combined Annotation-Dependent Depletion
CNAG: Centro Nacional de Análisis Genómico
gnomAD: Genome Aggregation Database
HANAC: hereditary angiopathy with nephropathy, aneurysms, and muscle cramps
OMIM: Online Mendelian Inheritance in Man
PVL: periventricular leukoencephalopathy
THR: triple-helix region
WES: whole-exome sequencing
